# Epidemiology of Cholesteatoma in the UK Biobank

**DOI:** 10.1111/coa.14257

**Published:** 2024-12-04

**Authors:** Emma Wilson, Barbara Anne Jennings, Mizanur Khondoker, Carl M. Philpott, Peter Prinsley, Daniel S. Brewer

**Affiliations:** ^1^ Norwich Medical School University of East Anglia Norwich UK; ^2^ ENT Department James Paget University Hospitals NHS Foundation Trust Great Yarmouth Norfolk UK; ^3^ Norwich and Norfolk University Hospital Norfolk UK; ^4^ Earlham Institute, Norwich Research Park Norwich UK

**Keywords:** cholesteatoma, epidemiology, hearing loss, middle ear, otitis media

## Abstract

**Objectives:**

To identify factors associated with cholesteatoma in a large UK cohort. Although some risk factors are frequently reported (male sex, history of chronic otitis media), other associations require further evidence (deprivation, smoking).

**Design and Setting:**

Briefly, 1140 cholesteatoma cases from UK BioBank were compared to 4551 non‐cholesteatoma middle ear disease and 493 832 ear disease‐free controls. Adjusted odds ratios were calculated for demographic factors including age, sex, ethnicity, deprivation and smoking status with logistic regressions. Odds ratios for overlapping ICD‐10 codes are also calculated.

**Results:**

Cholesteatoma was significantly associated with sex (Adjusted odds ratio (AOR) for males = 1.33, 95%CI = [1.179–1.491]), age (AOR = 1.02, 95%CI = [1.011–1.026]) and deprivation (AOR = 1.08, 95%CI = [1.059–1.097]) compared to ear disease‐free controls (*p* < 0.001). Age and deprivation distributions for cholesteatoma and non‐cholesteatoma ear disease were similar. Although there was no significant association with smoking status, cholesteatoma was significantly associated with the ICD‐10 code mental and behavioural disorders due to tobacco use (OR = 2.34, *p* < 0.001, 95%CI = [1.942, 2.813]). Cholesteatoma was also strongly associated with a wide range of inflammatory middle ear conditions and chronic sinus inflammation, suggesting an increased susceptibility to inflammation of the upper airways.

**Conclusion:**

This study shows a large overlap between cholesteatoma and non‐cholesteatoma ear disease in terms of numbers and demographics, with sex being a key factor distinguishing between the two, suggesting that there are both common and distinct associated factors.

**Summary**

- We present the first epidemiological study of cholesteatoma using data from UK BioBank.
- We identified 1140 cholesteatoma cases from the UK Biobank cohort. These cases were compared with two different control groups: matched ear disease controls and ear disease‐free controls.
- Demographic and ICD‐10 data are comprehensively summarised from our case–control study.
- Cholesteatoma was significantly associated with male sex, increasing age and greater deprivation.
- Associations were shared between cholesteatoma and other inflammatory middle ear diseases, but male sex was a risk factor distinguishing these groups.

## 1 Introduction

### 1.1 Background

Cholesteatoma is an invasive, non‐cancerous cyst of the middle ear whose expansion and destruction of surrounding bone results in progressive hearing loss, discharge and potentially serious intracranial complications including meningitis and abscess [1]. The only known treatment is surgical excision, which itself can contribute to hearing loss. Cholesteatoma incidence varies between populations but is reported up to 9.81 per 100 000 adults per year in Finland [2]. Not much is known about why cholesteatoma forms but several risk factors have been identified, including male sex [1], family history [3, 4] and certain developmental disorders affecting cranial morphology, including Turner syndrome and cleft palate [5]. While these risk factors are well known, associations with deprivation [2, 6, 7] and smoking [8] have occasionally been reported but require further investigation. Meanwhile, increased incidence in white over non‐white populations is often referenced [1, 9, 10, 11], but original epidemiological data supporting this is scant. Furthermore, cholesteatoma incidence has been shown to differ between populations and over time.

The UK BioBank (UKBB) is a database and large research resource that includes in depth health and genetic data from a cohort of ~500 000 participants recruited from the UK between 2006 and 2010. Hospital inpatient data is available for the whole cohort, with primary care data available for less than half of the participants [12]. Data about health and lifestyle were collected by questionnaire at recruitment and UKBB use algorithms to define health outcomes from self‐reported health information, hospital inpatient data and death data.

Greater understanding of cholesteatoma, its risk factors, and relationships to other middle ear disease are needed to inform study design for the discovery of preventative measures or non‐surgical treatments.

### 1.2 Aims and Objectives

This study aims to use retrospective data about UKBB participants to characterise lifetime prevalence and demographics related to cholesteatoma amongst a cohort aged > 52 years. We also compare cholesteatoma directly to non‐cholesteatoma middle ear disease (NC‐MED) to determine whether risk factors and associations are shared or unique. In addition, we aimed to compare these results to publicly available statistics from an equivalent Finnish Biobank, FinnGen (https://www.finngen.fi/en). This will be useful for other researchers considering the use of this biobank for the study of cholesteatoma and related conditions.

## 2 Methods

### 2.1 Study Design and Setting

This is a retrospective case–control study of data from UKBB participants (application ID 61632) who were drawn from the UK population between 2006 and 2010 aged 40–69 at recruitment. All participants provided informed consent through electronic signature at their baseline assessment.

UKBB has obtained Research Tissue Bank (RTB) approval from its ethics committee that covers our use of downloaded data. The Research Ethics Committee (REC) approval number for UKBB is 16/NW/0274. Researchers can acquire UKBB data by registering for access: (https://www.ukbiobank.ac.uk/enable‐your‐research/register). Publicly available demographic data and statistical results from FinnGen release 9 were accessed via Risteys (https://r9.risteys.finngen.fi/endpoints/H8_CHOLEASTOMA) in June 2023.

### 2.2 Participants

From the UKBB cohort, we identified individuals as having (or very likely having had) cholesteatoma. In this study, we use UKBB demographic data, including ethnicity, sex, birth year and deprivation and clinical classification codes from health records, to characterise cholesteatoma prevalence and associated diseases amongst this cohort.

### 2.3 Variables

Variables examined in the Biobank and FinnGen data included:

- Age, sex and ethnicity as given in response to a survey at recruitment.
- Smoking status, compiled from survey results, indicating whether participants have ever smoked.
- Townsend deprivation index (a measure of postcode deprivation combining employment rate, car and house ownership and household crowdedness [13]).
- International classification of diseases (ICD‐10) codes from medical records, which reflect participants' medical histories prior to recruitment and up to the date of download.

The UKBB participants were divided into three groups (Table S1 contains full codes and justifications):

- The cholesteatoma group, defined by ICD‐10 and operative (OPC‐4) codes which strongly suggest a history of cholesteatoma, including chronic mastoiditis, recurrent cholesteatoma, mastoidectomy and cholesteatoma itself.
- A NC‐MED group, who have any middle ear disease code or otalgia, otorrhea or otitis externa. Although not strictly a middle ear disease, otitis externa involves inflammation affecting the ear drum. Given the role of inflammation in establishment of cholesteatoma, we include this in our definition.
- A control group free of middle ear disease.

### 2.4 Study Size

The study size was determined by the availability of eligible data within the Biobank and FinnGen databases.

### 2.5 Statistical Analyses

#### 2.5.1 Logistic Regressions for Demographic Factors

Associations with demographic factors were tested by logistic regression of cholesteatoma status for cholesteatoma (*n* = 1140) versus control (*n* = 493 832) and cholesteatoma versus NC‐MED (*n* = 4551). Logistic regression models for the binary outcome of cholesteatoma status were fit on age, sex, smoking status, deprivation and ethnicity using the *fitglm* function with binomial error distribution and logit link function in MATLAB R2020b [14]. Adjusted odds ratios (AORs) were obtained for all covariates; 2884 individuals with missing smoking data were excluded from these regressions.

Sensitivity analysis was conducted to assess the robustness of the results to large case–control imbalance. We calculated unadjusted odds ratios for each variate using data matched for the remaining variates with a ratio of 1:5 cases to controls. Matching was performed using the package MatchIt (version 4.4.0) [15] in R 4.1.3 [16]. Exact matching was used for sex and ethnicity and propensity score‐based nearest neighbour matching for all remaining covariates. This resulted in good balance of all covariates across cases and controls without failing to match any cases (Table S2).

#### 2.5.2 Logistic Regressions for Disease‐Disease Associations

To investigate associations between cholesteatoma and other ICD‐10 codes, we performed pairwise logistic regression on cholesteatoma status against ICD‐10 code presence/absence. ICD‐10 codes were first collapsed to their parent category (e.g., H72.1 becomes H72). Non‐relevant codes were removed, such as those for medications or accidents (codes starting V, X, Y, Z, S, T or R). Presence–absence of a remaining 1312 ICD‐10 parent codes were tested separately for association with cholesteatoma using the MATLAB *fitglm* function with age and sex as covariates. All *p* values were Bonferroni corrected to adjust for the number of codes tested. Parent codes varied greatly in the number of cases present amongst the full, unmatched data (*n* = 1–151 022). All cholesteatoma cases (*n* = 1151) were compared to the full set of controls with and without other ear disease (*n* = 501 256). Participants with missing smoking data were retained in this analysis.

#### 2.5.3 Cox Hazard Regression for Comparison to FinnGen

The FinnGen comparison data include Cox hazard ratios for ICD‐10 codes before and after cholesteatoma. To compare the UKBB data to FinnGen, we performed Cox hazard regression on the set of codes with significant hazard ratios in FinnGen. UKBB cholesteatoma cases with time to event information (*n* = 650) were compared to ear disease‐free controls (*n* = 496 667) as in Risteys. Age and sex were used as covariates.

**FIGURE 1:**
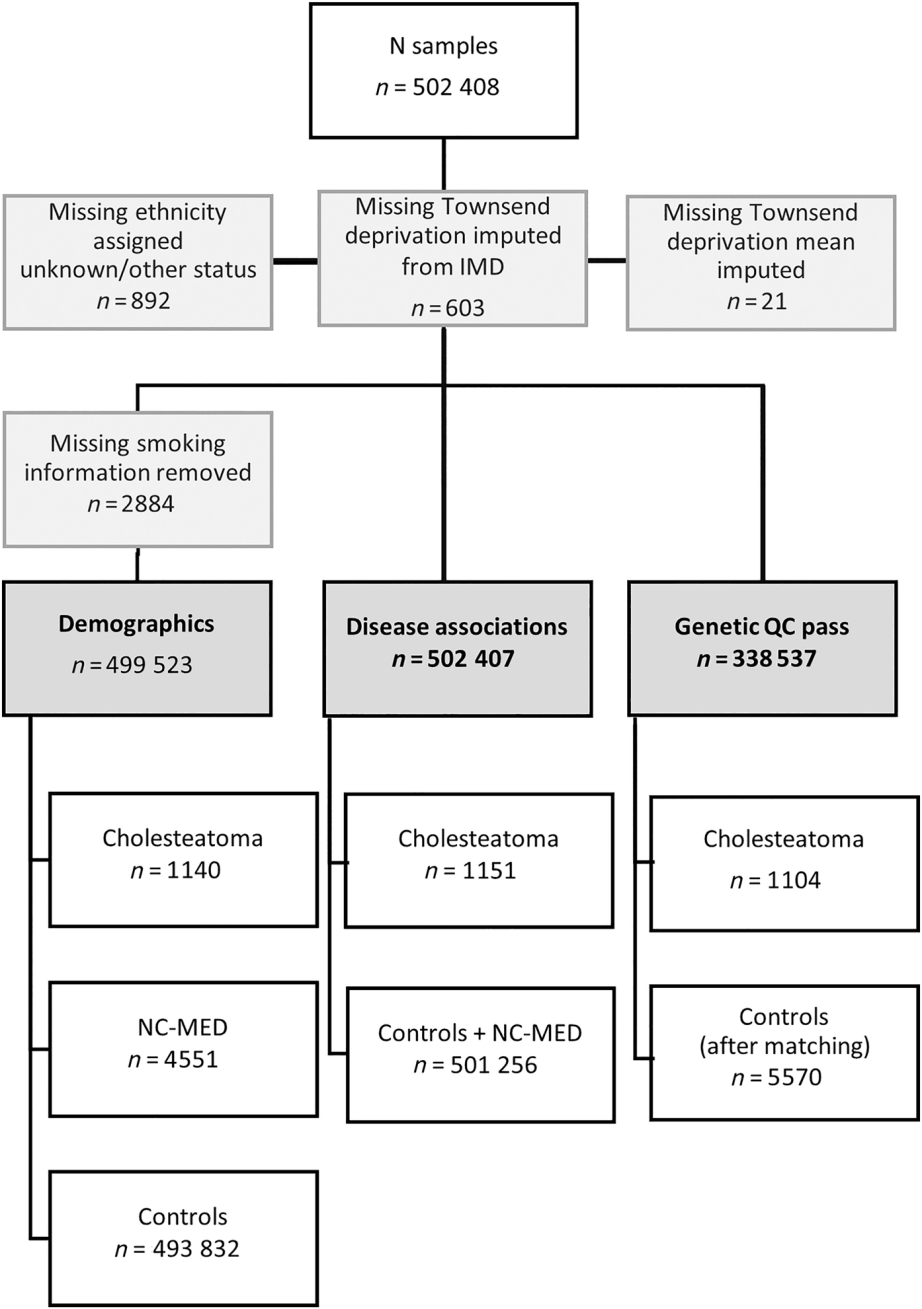
Participant numbers and missingness information for cases and controls. One participant was excluded for missing all covariates. Deprivation was imputed from indices if multiple deprivation (IMD) where available. Where not available, Townsend deprivation index was mean imputed. 2884 cases with missing smoking data were excluded from demographic analysis.

Figure 1 shows handling of missing data.

## 3 Results

### 3.1 Descriptive Data

#### 3.1.1 Demographics of Cholesteatoma and Non‐Cholesteatoma Middle Ear Disease

Total prevalence of cholesteatoma in UKBB was 0.22%, corresponding to approximately 1 in 500 people. Prevalence was higher in males with a male/female ratio of 1:1.35. In comparison, the prevalence in FinnGen was 0.38% with a male/female ratio of 1:1.67. The median age of cholesteatoma and NC‐MED cohorts in UKBB was 73 (IQR = 12 for both), while the median age of controls was 72 (IQR = 13). The median deprivation index of the cholesteatoma cohort was −1.40 (IQR = 5.14); NC‐MED −1.70 (IQR = 4.82); and the controls −2.14 (IQR = 4.18), where the higher scores indicate most deprivation.

### 3.2 Main Results

**FIGURE 2:**
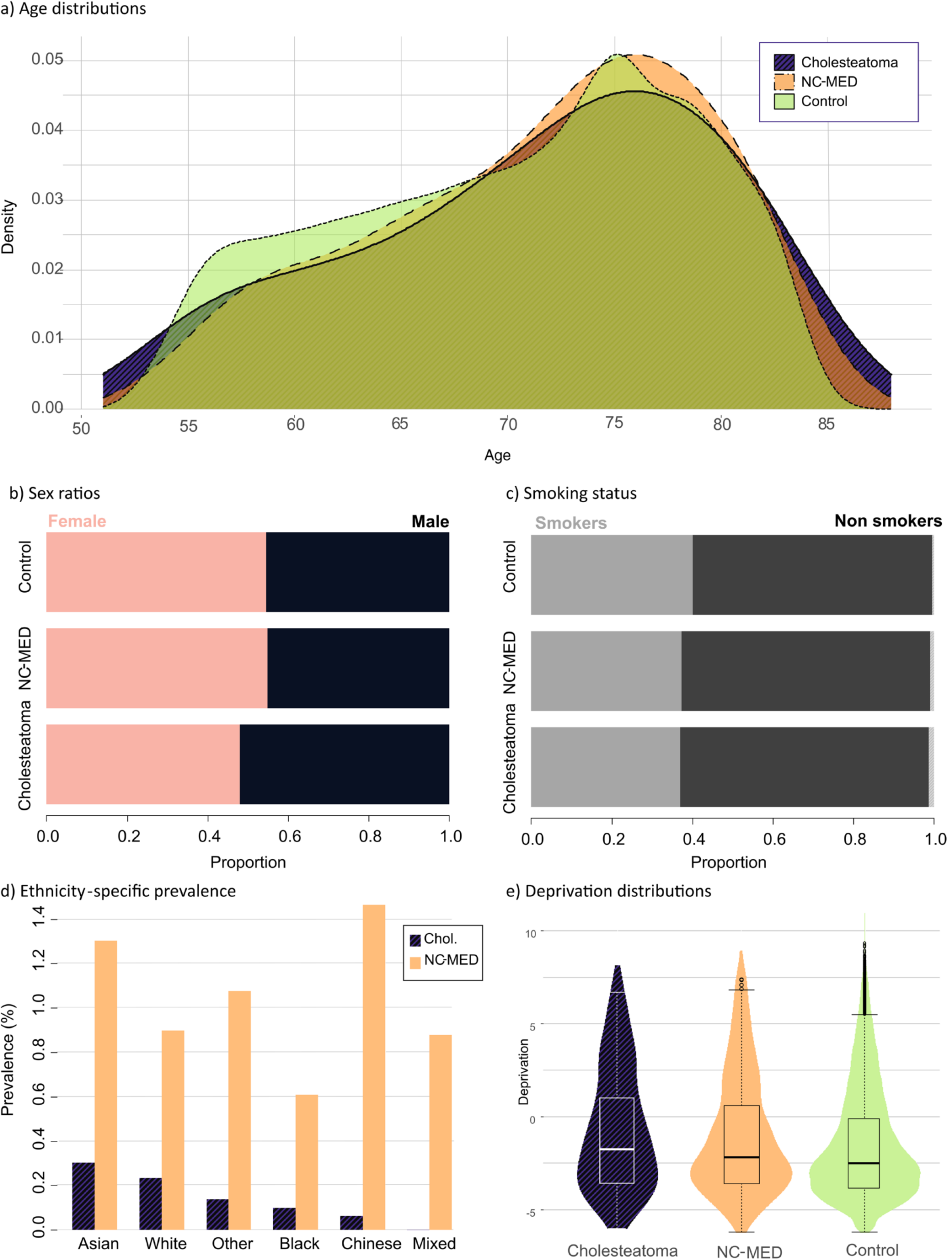
Demographics of unmatched cholesteatoma cases, non‐cholesteatoma middle ear disease (NC‐MED) and ear disease‐free controls showing (a) age distributions, (b) sex ratios, (c) smoking status, (d) prevalence of cholesteatoma and non‐cholesteatoma ear disease by ethnicity and (e) Townsend deprivation index distribution. Plots generated in R using ggplot2 package.

Significant associations with cholesteatoma incidence were found for sex (male AOR = 1.33, *p* < 0.001, 95%CI = [1.179, 1.491]), deprivation (AOR = 1.08, *p* < 0.001, 95%CI = [1.059–1.097]), age (AOR = 1.02, *p* < 0.001, 95%CI = [1.011, 1.026]), Black ethnicity (AOR = 0.35, *p* = 0.0035, 95%CI = [0.175, 0.71]) and other/unknown ethnicity (AOR 0.48, *p* = 0.042, 95%CI = [0.241, 0.973]) (Table 1, Figure 2d). The ORs obtained in sensitivity analysis for demographic factors generally agreed with the AORs obtained from the unmatched data except for the other/unknown ethnicity, showing that imbalance did not greatly affect the results (Table S3).

**TABLE 1:** Descriptive and inferential statistics of cholesteatoma in the UK Biobank.

				Versus disease‐free controls			Versus other ear disease		
	Prevalence (%)	*N* cases	*N* total	AOR	95%CI	*p*	AOR	95%CI	*p*
Total	0.22	1151	502 407						
**Female**	**0.20**	**533**	**271 839**						
Male	0.27	607	227 684	1.33	1.179, 1.491	< 0.001	1.30	1.142, 1.486	< 0.001
**White**	**0.23**	**1093**	**470 982**						
Mixed	0	0	2940	0.000	0, Inf	1	0.00	0, Inf	1
Asian	0.31	30	9769	1.237	0.857, 1.787	0.26	0.84	0.56, 1.268	0.41
Black	0.10	8	7998	0.352	0.175, 0.71	0.0035	0.60	0.28, 1.28	0.19
Chinese	0.06	1	1569	0.287	0.04, 2.041	0.21	0.17	0.023, 1.283	0.086
Other/unknown	0.13	8	6265	0.484	0.241, 0.973	0.042	0.45	0.212, 0.935	0.033
**Non‐smokers**	**0.21**	**420**	**200 812**						
Smokers	0.24	720	298 711	1.06	0.934, 1.194	0.38	0.98	0.856, 1.128	0.80
**Deprivation**	**—**	**—**		**1.08**	1.059, 1.097	**< 0.001**	**1.02**	1.001, 1.042	**0.040**
**Age**	**—**	**—**		**1.02**	1.011, 1.026	**< 0.001**	**1**	0.99, 1.007	**0.69**

*Note*: Prevalence and number of cases by demographic is shown alongside the total number of each demographic within the entire UK BioBank cohort. Adjusted odds ratios (AORs) and *p* values acquired from logistic regression of demographic factors on case status compared to a middle ear disease‐free control cohort and a non‐cholesteatoma ear disease cohort are also shown. Bold indicates the comparison category.

Comparing cholesteatoma to NC‐MED shows a similar male bias with an AOR of 1.30 (*p* < 0.001, 95%CI = [1.142, 1.486]), meaning the cholesteatoma group differs from NC‐MED about as much as it differs from the controls (AOR = 1.33; Cases vs. control; Figure 2b). The cholesteatoma and NC‐MED ear disease cohorts do not differ significantly in age or smoking status (*p* > 0.05) (Figure 2a,c). Deprivation was significantly associated with cholesteatoma but to a lesser extent than when compared to healthy ears (AOR 1.02, *p* = 0.040, 95%CI = [1.001, 1.042]) and the NC‐MED group has a more similar distribution of deprivation to the cholesteatoma group than the controls (Figure 2e).

### 3.3 ICD‐10 Associations With Cholesteatoma

**FIGURE 3:**
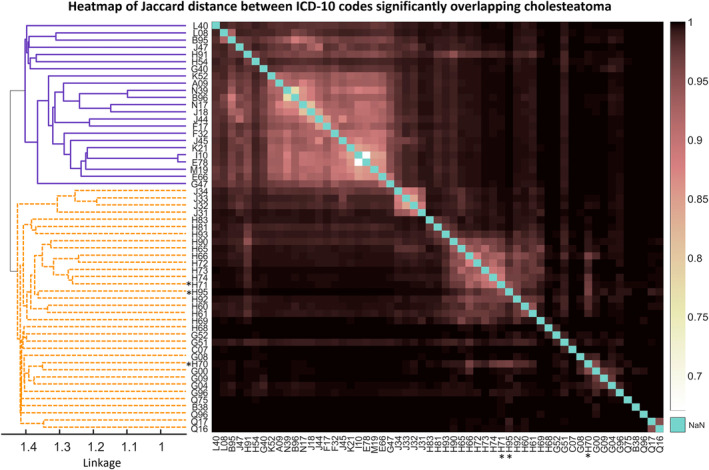
Heatmap of Jaccard distance between ICD‐10 codes where odds ratio adjusted *p* value for cholesteatoma association < 0.05. Colour scale indicates Jaccard distance with diagonals coloured blue. Hierarchical clustering using unweighted average distance/UPGMA (left) shows two main groups of disease codes: Common diseases (dark blue, solid) and sinus/middle ear infections with their rare complications (orange, dashed). Table 2 contains ICD‐10 code full names and statistics for all codes with adjusted *p* value < 0.05. Codes marked with an asterisk (*) have child codes used in the definition of the case group.

Briefly, 56 ICD‐10 codes were significantly associated with cholesteatoma after Bonferroni multiple testing correction (*p* < 0.05; Table 2). Hierarchical clustering of Jaccard distance between these codes reveals two main groups: common conditions, including chronic obstructive pulmonary disease (OR = 2.03, 95%CI = [1.653, 2.495]), disorders of lipoprotein metabolism and other lipidaemia (OR = 1.48, 95%CI = [1.28, 1.703]) and gastro‐oesophageal reflux (OR = 1.51, 95%CI = [1.288, 1.771]); and diseases of the sinuses and middle ear and their complications (Figure 3).

**TABLE 2:** Table of ICD‐10 codes significantly associated with cholesteatoma.

Code		*N*	*N* with cholesteatoma	Odds ratio	95%CI	Adjusted *p*	% Overlap
H66	Suppurative and unspecified otitis media	1275	263	144.54	124.259, 168.125	< 0.001	12.16
H72	Perforation of tympanic membrane	1504	193	76.07	64.549, 89.646	< 0.001	7.84
H73	Other disorders of tympanic membrane	570	142	163.63	133.92 9, 199.912	< 0.001	8.99
H74	Other disorders of middle ear and mastoid	695	219	242.12	203.651, 287.867	< 0.001	13.46
H92	Otalgia and effusion of ear	911	126	78.22	64.143, 95.383	< 0.001	6.51
H95*	Postprocedural disorders of ear and mastoid NEC (included in cholesteatoma definition)	162	117	1249.85	880.243, 1774.661	< 0.001	9.78
H90	Conductive and sensorineural hearing loss	2961	152	25.81	21.65, 30.774	< 0.001	3.84
H70*	Mastoiditis	181	153	2809.55	1865.538, 4231.256	< 0.001	
H65	Nonsuppurative otitis media	1587	100	30.80	24.924, 38.073	< 0.001	3.79
H91	Other hearing loss	10 920	213	9.94	8.514, 11.608	< 0.001	1.80
H60	Otitis externa	1048	75	34.97	27.429, 44.577	< 0.001	3.53
H61	Other disorders of external ear	1741	88	23.73	18.972, 29.683	< 0.001	3.14
H93	Other disorders of ear NEC	1874	45	10.65	7.875, 14.407	< 0.001	1.51
G51	Facial Nerve disorders	1848	42	10.10	7.391, 13.793	< 0.001	1.42
G00	Bacterial meningitis NEC	186	16	41.78	24.919, 70.056	< 0.001	1.21
F17	Mental and behavioural disorders due to tobacco use	24 886	127	2.34	1.942, 2.813	< 0.001	0.49
J32	Chronic sinusitis	4446	41	4.09	2.993, 5.6	< 0.001	0.74
H83	Other diseases of inner ear	1591	22	6.04	3.946, 9.235	< 0.001	0.81
Q16	Congenital malformations of ear causing impairment of hearing	25	4	82.05	28.032, 240.146	< 0.001	0.34
G96	Other disorders of central nervous system	562	12	9.55	5.371, 16.968	< 0.001	0.71
H69	Other disorders of Eustachian tube	317	9	12.99	6.678, 25.288	< 0.001	0.62
I10	Essential (primary) hypertension	151 022	478	1.53	1.355, 1.737	< 0.001	0.32
J44	Chronic obstructive pulmonary disease	21 261	103	2.03	1.653, 2.495	< 0.001	0.46
J45	Asthma	47 150	172	1.71	1.454, 2.013	< 0.001	0.36
Q17	Other congenital malformations of ear	42	3	36.73	11.312, 119.238	< 0.001	0.25
G04	Encephalitis, myelitis and encephalomyelitis	426	8	8.06	3.991, 16.26	< 0.001	0.51
B96	Sequelae of other and unspecified infectious and parasitic diseases	18 715	84	1.92	1.534, 2.401	< 0.001	0.42
J34	Other disorders of nose and nasal sinuses	9626	49	2.24	1.68, 2.984	< 0.001	0.46
F32	Other depressive episodes	29 778	110	1.74	1.425, 2.114	< 0.001	0.36
Q75	Other congenital malformations of skull and face bones	17	2	59.42	13.526, 261.048	< 0.001	0.17
E78	Disorders of lipoprotein metabolism and other lipidaemias	77 039	262	1.48	1.28, 1.703	< 0.001	0.34
J47	Bronchiectasis	5742	34	2.46	1.741, 3.465	< 0.001	0.50
K21	Gastro‐oesophageal reflux disease	54 508	183	1.51	1.288, 1.771	< 0.001	0.33
G40	Epilepsy	6849	37	2.33	1.674, 3.231	< 0.001	0.46
K52	Other noninfective gastroenteritis and colitis	25 678	96	1.68	1.363, 2.074	0.002	0.36
L40	Psoriasis	5499	31	2.40	1.68, 3.438	0.002	0.47
H68	Eustachian salpingitis and obstruction	28	2	33.08	7.82, 139.896	0.003	0.17
H54	Visual impairment including blindness	2813	20	2.92	1.869, 4.548	0.003	0.51
N17	Acute renal failure	22 036	92	1.69	1.357, 2.094	0.003	0.40
B95	Streptococcus and staphylococcus as the cause of diseases classified to other chapters	8860	44	2.07	1.529, 2.801	0.003	0.44
G52	Disorders of other cranial nerves	158	4	10.83	4.005, 29.299	0.004	0.31
H81	Disorders of vestibular function	3088	20	2.80	1.793, 4.359	0.008	0.47
J18	Bronchopneumonia, unspecified	26 445	103	1.61	1.309, 1.974	0.008	0.37
L08	Other local infections of skin and subcutaneous tissue	3198	21	2.71	1.759, 4.187	0.008	0.49
G47	Sleep disorders	11 734	53	1.89	1.43, 2.491	0.009	0.41
Q96	Turner syndrome	45	2	25.18	6.078, 104.301	0.011	0.17
C07	Malignant neoplasm of parotid gland	179	4	9.50	3.519, 25.657	0.012	0.30
E66	Obesity	35 634	122	1.53	1.267, 1.846	0.012	0.33
G08	Intracranial and intraspinal phlebitis and thrombophlebitis	103	3	13.29	4.205, 42.001	0.014	0.24
M19	Other arthrosis	45 012	150	1.48	1.239, 1.757	0.016	0.33
A09	Other gastroenteritis and colitis of infectious and unspecified origin	19 953	76	1.66	1.318, 2.102	0.025	0.36
G09	Sequelae of inflammatory diseases of central nervous system	104	3	11.97	3.788, 37.821	0.031	0.24
N39	Other disorders of urinary system	38 025	128	1.49	1.24, 1.798	0.032	0.33
J31	Chronic rhinitis, nasopharyngitis and pharyngitis	1743	13	3.22	1.862, 5.582	0.038	0.45
J33	Nasal polyp	4670	26	2.28	1.545, 3.374	0.045	0.45
B38	Coccidioidomycosis	4	1	119.72	12.384, 1157.385	0.047	0.09

*Note*: Association tests were performed using logistic regression with age and sex as covariates. Codes are collapsed to parent code, and the ICD‐102019 description is given. Codes marked with an asterisk (*) were included in the case definition.

The strongest associations (all *p* values < 0.001) were with other disorders of middle ear and mastoid (OR = 242.12, 95%CI = [203.651, 287.867]), other disorders of tympanic membrane (OR = 163.63, 95%CI = [133.92 9, 199.912]), suppurative and unspecified otitis media (OR = 144.54, 95%CI = [124.259, 168.125]), otalgia and effusion of the ear (OR = 78.22, 95%CI = [64.143, 95.383]) and perforation of the tympanic membrane (OR 76.07, 95%CI = [64.549, 89.646]). The overlap with cholesteatoma for each of these conditions was 100–263 persons. Otitis externa (OR = 34.97, *p* < 0.001, 95%CI = [27.429, 44.577]) and other diseases of inner ear (OR = 6.04, *p* < 0.001, 95%CI = [3.946, 9.235]) were also associated with cholesteatoma. Known complications of cholesteatoma were also strongly associated, including sensorineural and conductive hearing loss (OR = 25.81, 95%CI = [21.65, 30.774]), other hearing loss (OR = 9.94, 95%CI = [8.514, 11.608]), facial nerve disorders (OR = 10.10, 95%CI = [7.391, 13.793]) and bacterial meningitis (OR = 41.78, 95%CI = [24.919, 70.056]).

The most strongly associated non‐ear code not known to be a cholesteatoma complication was F17, mental and behavioural disorders due to tobacco use with an OR of 2.34 (*p* < 0.001, 95%CI = [1.942, 2.813]), followed by chronic sinusitis, with an OR of 4.09 (*p* < 0.001). Several other respiratory conditions are represented including chronic obstructive pulmonary disease, asthma and chronic rhinitis with and without nasal polyps. Some congenital anomalies affecting the ear and head (Q17, Q16, Q75, Q96) were also strongly associated with cholesteatoma (OR = 33.58–83.24, *p* ≤ 0.011), although the number of overlapping cases was small (*n* = 2–4).

### 3.4 Comparison to FinnGen Significant Associations

Diseases occurring before cholesteatoma with significantly increased hazard ratios in both biobanks were otosclerosis and sleep apnoea (Table 3). Hazards of otosclerosis, epilepsy and chronic kidney disease were significantly increased after cholesteatoma in both biobanks.

**TABLE 3:** Comparison of UKBB odds ratios and hazard ratios to FinnGen.

		FinnGen		UKBB		UKBB	
		HR [95%CI]	*p*	OR [95%CI]	*p* (uncorrected)	HR [95%CI]	*p*
Before cholesteatoma	Otosclerosis	6.80 [4.14, 11.18]	< 0.001	5.18 [2.144, 12.552]	< 0.001	7.98 [7.98, 31.998]	0.003
	Sudden idiopathic hearing loss	2.93 [1.62, 5.31]	< 0.001	9.94 [8.514, 11.608]^a^	< 0.001	0 [0, 8.65 × 10^171^]	0.974
	Arthrosis^b^	0.72 [0.7, 0.91]	0.0062			1.12 [1.12, 1.545]	0.974
	Pre‐eclampsia or eclampsia^c^	1.80 [1.09, 2.96]	0.022				
	Gonarthrosis	0.71 [0.53, 0.96]	0.025	0.98 [0.796, 1.215]	0.87	0.78 [0.78, 1.327]	0.356
	Coxarthrosis	0.59 [0.37, 0.93]	0.025	0.96 [0.739, 1.259]	0.79	0.88 [0.88, 1.711]	0.708
	Sleep apnoea	1.38 [1.04, 1.83]	0.028	1.88 [1.43, 2.491]^d^	< 0.001	2.1 [2.1, 4.065]	0.029
	Pain^e^	1.18 [1.01, 1.38]	0.036				
After cholesteatoma	Otosclerosis	6.06 [3.41, 10.76]	< 0.001	5.18 [2.144, 12.552]	< 0.001	8.7 [8.7, 34.952]	0.002
	Vascular dementia	5.02 [2.36, 10.68]	< 0.001	3.66 [0.217, 2.105]	0.19	0.93 [0.93, 3.737]	0.923
	Epilepsy	1.79 [1.09, 2.93]	0.021	1.47 [1.869, 4.548]	< 0.001	2.85 [2.85, 4.733]	< 0.001
	Chronic kidney disease	1.77 [1.08, 2.92]	0.025	1.43 [1.119, 1.812]	0.0041	1.5 [1.5, 2.09]	0.017
	Iron deficiency anaemia	1.74 [1.07, 2.82]	0.026	1.44 [1.136, 1.829]	0.0027	1.17 [1.17, 1.746]	0.441
	Varicose veins	0.59 [0.35, 0.98]	0.041	1.061 [0.776, 1.45]	0.71	1.01 [1.01, 1.885]	0.964

*Note*: Table showing Cox hazard ratios computed by Risteys from FinnGen data where *p* < 0.05 and equivalent Cox hazard ratios and *p* values computed for UKBB data. Also shown are the uncorrected *p* values and odds ratios drawn from logistic regressions for the closest equivalent ICD‐10 codes. Risteys phenotypes are as follows.

^a^ Showing H91 for UKBB OR, parent code of sudden idiopathic hearing loss (H91.2).

^b^ Arthrosis combines parent categories M15‐M19.

^c^ Combination of eclampsia (O15) and pre‐eclampsia (O14). No overlap with cases in UKBB.

^d^ Showing G47 for UKBB OR, parent code of sleep apnoea (G47.3).

^e^ Hybrid category of several ICD‐10 codes involving pain in FinnGen, not tested in UKBB.

## 4 Discussion

### 4.1 Key Results

Here we present an epidemiological study of cholesteatoma in the UKBB to establish demographic factors associated with cholesteatoma in this cohort and to identify associated diseases using ICD‐10 codes. We also compared these results with summary analyses performed on data from the Finnish biobank FinnGen. Our study replicated some demographic risk factors for cholesteatoma identified in previous studies. We also found a large overlap between cholesteatoma and other middle ear diseases, and the demographics of these groups are similar except for sex and ethnicity.

### 4.2 Associations With Sex, Deprivation and Smoking

Male sex was an important risk factor for cholesteatoma compared to disease‐free controls (AOR = 1.33, 95%CI = [1.179–1.491]). A male predominance is well established in the literature and the sex ratios of 1:13 females to males in UKBB and 1:1.67 in FinnGen are similar to the reported range [2, 17]. Both cholesteatoma and NC‐MED were associated with higher deprivation, with cholesteatoma associated with the highest deprivation (median − 1.40, −1.70 and −2.14, respectively). Deprivation is associated with risk behaviours such as smoking [18, 19], and the most disadvantaged are both more likely to require healthcare and less likely to access it [20]. These factors may contribute to greater rates of ear disease and cholesteatoma. However, it is also possible that increased deprivation results from cholesteatoma due to the economic impact of hearing loss. Increased odds of several common diseases found in this study may also reflect generally poorer health in the cholesteatoma group. Meanwhile, the odds ratio associated with age for both cholesteatoma and NC‐MED is likely due to older participants having had longer to develop any disease.

Rates of cholesteatoma have also been reported to vary with ethnicity [1], although original epidemiological data is rarely presented. Both rates of cholesteatoma and NC‐MED varied between ethnicities in this analysis, although the only consistent significant difference was for the Black ethnicity, who had reduced odds of both cholesteatoma and NC‐MED. The Chinese and Asian groups also had increased odds of NC‐MED, but the sample sizes may have been too small to properly quantify their risk of cholesteatoma.

Although smoking was not significantly associated with cholesteatoma according to the survey results, the disease code F17 mental and behavioural disorders due to use of tobacco was (OR 2.34, 95%CI = [1.942, 2.813]). This suggests that infrequent or discontinued smoking affects risk of cholesteatoma less than heavy smoking. This confirms the results of Kaylie et al. [8], where they found a higher rate of cholesteatoma and more severe disease in smokers with chronic ear problems, but that former smokers had similar outcomes to non‐smokers > 5 years after smoking cessation. Smoking may increase susceptibility to ear disease by impairing mucociliary function [8], which may also impact cholesteatoma risk.

### 4.3 Sex Distinguishes Cholesteatoma From Other Middle Ear Disease

A key finding was that the cholesteatoma and NC‐MED cohorts were more similar to each other than the controls on several characteristics. Sex ratio was a key difference distinguishing cholesteatoma from NC‐MED, with the AOR for cholesteatoma being similar when compared to NC‐MED or controls (AOR = 1.3 vs. NC‐MED; AOR = 1.33 vs. controls). Furthermore, sex ratio did not differ significantly between middle ear disease and the control group (*p* = 0.49; Table S4). This means that either males are not at increased risk of general ear disease, only of cholesteatoma; or that they are at increased risk of all forms of ear disease and at increased risk of sequelae, including cholesteatoma.

### 4.4 Cholesteatoma Is Significantly Associated With Other Inflammatory Ear and Respiratory Disease

In this study, we found a large overlap between the incidence of cholesteatoma and other middle ear disease. Many of these are likely consequences of cholesteatoma or related ear conditions, such as hearing loss, otorrhoea, tympanic perforation, mastoiditis, Eustachian tube disorders and several forms of otitis media. A history of ear disease, including chronic otitis media is well‐established risk factors in cholesteatoma: Kemppainen et al. [2] report history of otitis in 72.4% of cases, and Castle [21] reports common concurrent diseases including otic polyp and tympanosclerosis. Whether inflammation precedes cholesteatoma or is a symptom of it cannot be determined from this study design. However, children with repeated ear infection requiring ventilation tube insertion are at increased risk of cholesteatoma [22], implying that increased susceptibility to middle ear infection also increases cholesteatoma susceptibility, either directly or because both result from poor ear ventilation. Tympanic retraction is also a risk factor for cholesteatoma, though most retractions do not progress to cholesteatoma [23].

This study also found an association between cholesteatoma and chronic respiratory inflammation including rhinosinusitis and asthma. Chronic sinusitis may impact Eustachian tube function and ventilation of the middle ear, possibly contributing to cholesteatoma risk, or this may represent a general susceptibility to chronic inflammation of the airways.

Increased hazards of otosclerosis both before cholesteatoma in UKBB and FinnGen is a surprising finding as the co‐occurrence of these conditions is extremely rarely reported [24]. Otosclerosis is a disease of the labyrinth resulting from remodelling and overgrowth of the bone at the base of the stapes [25]. Though it is possible that a background of inflammation in the middle ear could increase risk of otosclerosis, these conditions rarely occur together and can easily be misdiagnosed. Because of the small number of overlapping cases (*n* = 5 in UKBB, 48 in FinnGen), this may be the cause of this association.

### 4.5 Known Complications of Cholesteatoma and a New Potential Association

We detected known rare complications of cholesteatoma such as facial nerve disorders (including Bell's palsy) and bacterial meningitis [26]. Similarly, other diseases of the nervous system such as encephalitis, myelitis and encephalomyelitis, and sequelae of inflammatory diseases of the central nervous system may also represent rare complications of cholesteatoma, which occur when the cyst erodes through the temporal bone. The risk of epilepsy following cholesteatoma was also significantly increased in both UKBB and FinnGen. Some of these cases could be a complication of cholesteatoma where there is intracranial involvement, and epilepsy has occasionally been described in this context [27, 28]. Alternatively, epilepsy itself may be a risk factor, as it is often associated with a high burden of comorbid disease [29].

We also detected increased odds of psoriasis and other local infections of skin and subcutaneous tissue with cholesteatoma. Cholesteatoma expresses several proteins, which are markers of inflammatory skin conditions, including members of the S100 protein family [30].

### 4.6 Study Limitations

The limitations of this study reflect the limitations of BioBank‐based observational studies. UKBB is biassed towards females, participants are wealthier and have fewer health conditions than the general population and is majority of white European background [31]. It is also likely that not all cholesteatoma cases could be identified due to the lack of ICD‐10 data prior to its introduction in 1995. We mitigated this by including likely cholesteatoma cases from related ICD‐10 and operative codes, but there are probably still missing cases, including childhood disease. Furthermore, no distinction has been made between congenital and acquired cholesteatoma in this study as they are impossible to distinguish from ICD codes.

Demographics such as deprivation were collected at the time of recruitment and may differ from the time of disease onset. Additionally, deprivation is based on postcode and may not reflect the status of the individual. Different demographic groups may also differ in their willingness to engage with a doctor, which will influence the apparent effect of these factors.

Numbers of cases and controls were highly imbalanced. To retain as much information as possible, unmatched controls were used for each analysis which can inflate *p* values. We performed sensitivity analysis for the demographic features using 1:5 matched controls to check that the effect of imbalance was not too severe. However, some ICD‐10 codes have even more extreme imbalance so should be interpreted with caution.

Conclusions about causality cannot be drawn from observational associations, for example, deprivation could be a sequela or a risk factor for the morbidity associated with cholesteatoma. Time to event information also reflects time of diagnosis, not necessarily the order of disease onset.

## 5 Conclusions

An increased risk of cholesteatoma is associated with male sex, older age and greater deprivation. There is a significant association between cholesteatoma and mental and behavioural disorders due to tobacco use, supporting heavy smoking as a risk factor. Although this study provides evidence of a higher incidence in white compared to non‐white populations, the sample size for non‐white ethnicities in the UKBB is too small to be conclusive.

Associations are shared between cholesteatoma and other inflammatory middle ear disease, but male sex was a major risk factor distinguishing these groups. Cholesteatoma overlaps significantly with other inflammatory middle ear conditions, but it is difficult to disentangle the factors contributing to ear disease in general from those contributing to cholesteatoma alone. Cholesteatoma was also positively associated with epilepsy and negatively associated with arthrosis in two biobanks. These relationships are unexplored in the literature and warrant further investigation.

Future studies of cholesteatoma should carefully consider their choice of control population (with middle ear disease or disease‐free ears), as such studies may address different questions about the epidemiology, genetics and pathology of cholesteatoma. This will enhance the ability to discover new risk factors and pathological features and shed further light on an otherwise poorly understood but important cause of hearing loss.

## Author Contributions

**Barbara Anne Jennings, Carl M. Philpott, Peter Prinsley, and Daniel S. Brewer:** conceptualization, funding acquisition, methodology, writing – review and editing (equal co‐authors). **Mizanur Khondoker:** methodology and statistical supervision, writing – review and editing (equal co‐authors). **Emma Wilson:** methodology and statistical analysis, investigation and formal analysis (lead), writing – original draft and editing (lead).

## Ethics Statement

UK Biobank has obtained Research Tissue Bank (RTB) approval from its ethics committee that covers our use of downloaded data. The Research Ethics Committee (REC) approval number for UK Biobank is 16/NW/0274.

## Conflicts of Interest

The authors declare no conflicts of interest.

### Peer Review

The peer review history for this article is available at https://www.webofscience.com/api/gateway/wos/peer‐review/10.1111/coa.14257.

## Supporting information


**Data S1.** Supporting Information.

**Data S2.** Supporting Information.

## Data Availability

The data that support the findings of this study are available in UK BioBank after registering for access: (https://www.ukbiobank.ac.uk/enable‐your‐research/register). Publicly available demographic data and statistical results from FinnGen release 9 can be accessed via Risteys (https://r9.risteys.finngen.fi/endpoints/H8_CHOLEASTOMA).
