# Supplementary material for: Epidemiology of Cholesteatoma in the UK Biobank
Source: Clin Otolaryngol. 2024 Dec 4;50(2):316–29. doi: 10.1111/coa.14257 (PMC11792432; doi:10.1111/coa.14257)
Supplement: Supplementary file 1 — Data S1. Supporting Information. [file COA-50-316-s002.docx]

# Supplementary Information

## Case selection criteria

**Table 1: Case inclusion and exclusion criteria**

| Filter | Code | Meaning | Rationale |
| --- | --- | --- | --- |
| Confirmed | H71 | Cholesteatoma of the middle ear | Unambiguous codes specify cholesteatoma |
|  | H95.0 | Recurrent cholesteatoma of postmastoidectomy cavity |  |
| Mastoiditis | H70.1 | Chronic mastoiditis | Chronic mastoiditis most likely to result from cholesteatoma |
|  | H70.9 | Unspecified mastoiditis |  |
| Suspected | D10.1 | Radical mastoidectomy NEC | Surgeries used primarily for treatment of cholesteatoma and few other conditions (see exclude filter) |
|  | D10.2 | Modified radical mastoidectomy |  |
|  | D10.6 | Revision mastoidectomy |  |
|  | D10.8 | Other specified exenteration of mastoid |  |
|  | D10.9 | Other unspecified exenteration of mastoid |  |
|  | D12.4 | Exploration of mastoid |  |
|  | D12.1 | Obliteration of mastoid |  |
|  | D12.2 | Atticotomy |  |
|  | D12.7 | Atticoantrostomy |  |
|  | D10.5 | Excision of lesion of mastoid | Probably indicates removal of cholesteatoma due to few other lesions affecting mastoid. |
| Suspected exclude | D33.3 | Benign neoplasm of cranial nerve | May indicate acoustic neuroma, alternative explanation for mastoidectomy. |
|  | H93.3 | Disorder of acoustic nerve |  |
|  | H70.0 | Acute mastoiditis | Possible cause for mastoidectomy without cholesteatoma. |
|  | H81.0 | Meniere disease |  |
|  | D02.3 | Middle ear carcinoma | Alternative explanation for excision of lesion of mastoid. |
|  | D38.5 | neoplasm of uncertain behaviour |  |
|  | C30.1 | Malignant neoplasm of middle ear |  |
|  | D16.9 | Benign neoplasm of bone and articular cartilage | To capture osteoma, alternative explanation for excision of lesion of mastoid. |
| Mastoid exclude | H65.0 | Acute serous otitis media | Exclude acute cases from mastoiditis group. |
|  | H65.1 | Other acute nonsuppurative otitis media |  |
|  | H66.0 | Acute suppurative otitis media |  |
| Other ear disease | H65-H75 | Diseases of middle ear and mastoid | Include any middle ear disease |
|  | H60 | Otitis externa | Inflammatory ear disease closely related to middle ear disease |
|  | H92 | Otalgia and effusion of ear | Ear pain and discharge suggests underlying ear disease |

An individual is considered a cholesteatoma case if they have one of the **confirmed** codes, one of the **suspected** codes but not a **suspected** **exclude** code, or one of the **mastoid** codes but not a mastoid **exclude** code. Suspected codes are OPC-4 (operative procedure) codes strongly suggestive of cholesteatoma treatment and exclude codes are possible alternative explanations. Chronic mastoiditis is assumed to indicate cholesteatoma unless it can be explained by acute otitis media.

The **other ear disease group** is defined by any individual with middle ear disease but not in the cholesteatoma group. The control group has no ICD-10 codes suggestive of other ear disease and is not in the cholesteatoma or middle ear group.

## Matching results

The MatchIt package uses propensity matching to select controls to produce the best balance of covariates across cases and controls. The propensity score is the likelihood of being a case based only on the covariates alone, in this case based on logistic regression. Controls are selected to produce similar propensity score distributions in the cases and controls. The selected method uses nearest neighbour to select controls with matching propensity score for cases, except for sex and ethnicity, which are exact matched. Because matching for this study was performed as part of a parallel genetic study, controls failing genetic quality control were not included in this analysis.

**Table 2: Balance summary for data propensity score matched using nearest neighbour and exact matched for ethnicity and sex**

|  | Standardised mean difference | | | Variance ratio | | |
| --- | --- | --- | --- | --- | --- | --- |
|  | Before | After | % Improvement | Before | After | Improvement |
| Propensity score | 0.370 | -0.0001 | 100.0 | 1.217 | 0.9996 | 99.8 |
| Sex | 0.1526 | 0.0000 | 100 |  |  |  |
| Ethnicity | -0.0122 | 0.0000 | 100 |  |  |  |
| Smoking | 0.0114 | -0.00054 | 88.82 |  |  |  |
| Age | 0.171 | -0.0374 | 78.1 | 0.949 | 1.0319 | 39.9 |
| Deprivation | 0.200 | 0.0154 | 92.3 | 1.1441 | 0.9886 | 91.5 |

## Sensitivity analysis for logistic regressions on matched data

**Table 3: Results of matched logistic regressions testing demographic factors**

| **Test** | **P value** | **OR** | **95% CI** |
| --- | --- | --- | --- |
| Age | **1.02x10^-5^** | **1.019** | **1.01-1.026** |
| Sex | **0.00028** | **1.267** | **1.115-1.439** |
| Deprivation | **3.57x10^-15^** | **1.079** | **1.058-1.1** |
| Smoking | 0.18 | 1.096 | 0.96-1.25 |
| White | **0.0056** | **1.592** | **1.167-2.174** |
| Mixed | -- | 1.000 | 1-1 |
| Asian | 0.98 | 0.994 | 0.674-1.495 |
| Black | **0.00033** | **0.270** | **0.132-0.552** |
| Chinese | 0.093 | 0.180 | 0.024-1.328 |
| Other | 0.85 | 10.93 | 0.436-1.987 |

Cholesteatoma cases were matched to middle ear disease-free controls with a 1:5 ratio using the same matching method, excluding the covariate being tested. As all other covariates are being controlled, the odds ratios acquired form this analysis are equivalent to the adjusted odds ratios acquired from the unmatched data. Significant results are highlighted in bold. The only association which was not significant in the sensitivity analysis was the Other/unknown ethnicity (AOR=1.14, p=0.68). The actual ethnic composition of this group is not known, therefore may vary from the primary analysis.

## Comparison of middle ear disease to controls

**Table 4: logistic regression results for comparison of middle ear disease to middle ear disease-free controls**

|  | Prevalence (%) | N | AOR | 95% CI | p |
| --- | --- | --- | --- | --- | --- |
| **Total** | **0.91** | **4589** |  |  |  |
| **Female** | 0.90 | 2,450 |  |  |  |
| Male | 093 | 2,139 | 1.02 | 0.963-1.083 | 0.49 |
| **Non-smokers** | 0.86 | 1,720 |  |  |  |
| Smokers | 0.94 | 2,831 | 1.07 | 1.004-1.135 | 0.038 * |
| **White** | 0.91 | 4,283 |  |  |  |
| Mixed | 0.88 | 26 | 0.97 | 0.656-1.427 | 0.87 |
| Asian | 1.31 | 130 | 1.46 | 1.222-1.746 | 3.23x10^-5^ *** |
| Black | 0.60 | 49 | 0.59 | 0.444-0.791 | 3.88x10^-4^ *** |
| Chinese | 1.46 | 23 | 1.71 | 1.133-2.592 | 0.011 * |
| Unknown | 1.06 | 78 | 1.06 | 0.825-1.36 | 0.65 |
| **Age** | -- | -- | 1.02 | 1.016-1.024 | 3.03x10^25^ *** |
| **Deprivation** | -- | -- | 1.06 | 1.046-1.065 | 2.28x10^-31^ *** |

Age and deprivation are a significant difference with similar odds ratios when comparing cholesteatoma to ear disease-free controls. Smoking was not significant when comparing cholesteatoma to disease-free controls, but the odds ratio was similar. This may be due to the smaller number of cholesteatoma cases than NC-MED. This supports the notion that cholesteatoma and middle ear disease share similar demographics except for sex. Meanwhile, the effect of ethnicity is difficult to quantify due to the small sample size. The Asian and Chinese groups have higher odds of NC-MED compared to controls but their odds of cholesteatoma are not significantly increased. This may be due to the smaller sample size, as with smoking.
